# How do channel densities and various time constants affect the dynamic gain of a detailed model of a pyramidal neuron?

**DOI:** 10.1186/1471-2202-14-S1-P419

**Published:** 2013-07-08

**Authors:** David Hofmann, Andreas Neef, Ilya Fleidervish, Michael Gutnick, Fred Wolf

**Affiliations:** 1 Bernstein Center for Computational Neuroscience Göttingen, Germany; 2 Max Planck Institute for Dynamics and Self-Organization, Göttingen, Germany; 3 Ben-Gurion University of the Negev, Israel; 4 Hebrew University of Jerusalem, Israel

## 

The axon initial segment (AIS) controls the transformation of dendrosomatic synaptic input into spike output and the backpropagation of action potentials into the dendrites due to its lower spike initiation threshold. Channel density and kinetics can both contribute to this low threshold. However, the nature of such threshold differences is unknown and topic of current debates [1-3].

Dynamical response properties give a constraint on the AIS function. Here we study the dynamical response properties of a detailed multi compartment NEURON [4] model that well reproduces the sodium concentration changes in the AIS and soma generated by action potential firing in a layer 5 pyramidal cell [2].

To study these properties, we inject different current stimuli into the soma. These are constant currents and Gaussian noise currents as studied in [5]. We vary the sodium and potassium channel densities at the axon initial segment as well as the sodium activation time constant *taum*. Furthermore, we study the influence of input current parameters as mean, variance and correlation time. We then calculate the dynamic rate response of a population of independent neurons. This is described at linear order by a filter function with frequency dependent gain as done by [5].

The f-I curves show that the neuron model under investigation is of type I. This holds true for all channel densities tested. The cut-off frequency appears insensitive to AIS channel density.
